# Bleaching of leaf litter accelerates the decomposition of recalcitrant components and mobilization of nitrogen in a subtropical forest

**DOI:** 10.1038/s41598-021-81206-7

**Published:** 2021-01-19

**Authors:** Takashi Osono, Syuntaro Hiradate, Satoru Hobara

**Affiliations:** 1 Faculty of Science and Engineering, Doshisha University, Kyoto, Japan; 2 Faculty of Agriculture, Kyushu University, Fukuoka, Japan; 3 College of Agriculture, Food and Environment Sciences, Rakuno Gakuen University, Ebetsu, Japan

**Keywords:** Ecosystem ecology, Forest ecology, Tropical ecology, Solid Earth sciences

## Abstract

Selective removal of lignin and other recalcitrant compounds, collectively registered as acid-unhyrolyzable residue (AUR), results in bleaching of leaf litter, but the importance of bleaching in decomposition processes on forest soil has not been fully evaluated. The aims of this study were to elucidate the occurrence of bleached area in decomposing leaf litter and to compare chemical composition between bleached and nonbleached portions in a subtropical forest in Japan. Field incubation of leaf litter was performed over an 18-month period with the litterbag method. The decomposition processes during the first 9 month were characterized by the relatively rapid mass loss and increase of bleached area, whereas the mass loss was slowed down and the bleached area decreased thereafter. Mass loss of leaf tissues was faster and AUR content was lower in bleached than in nonbleached portions, indicating the acceleration of mass loss in bleached leaf tissues by the selective decomposition of recalcitrant compounds. The decrease in carbonyl-C in the bleached portions was associated with the increase of extractable nitrogen. The results suggest that the bleaching plays a dominant role in the transformation and turnover of organic compounds and nitrogen in decomposing leaf litter.

## Introduction

Decomposition of leaf litter and the concomitant formation of soil organic matter and mobilization of nutrient is a crucial component of ecosystem functioning in forest soils^1,2^. Factors influencing the decomposition include climatic conditions, chemical quality, and decomposer organisms^3,4^, of which the composition of organic chemical components and essential nutrients exerts a primary control on the decomposition under particular climatic conditions. Previous studies have repeatedly documented the importance of recalcitrant compounds in leaf litter registered as acid-unhydrolyzable residue (AUR), including lignin, cutin, phenolic compounds, and condensed tannin, as the factor regulating decomposition rates and patterns of chemical changes during decomposition^5,6^. Because of the resistance of these compounds to microbial decomposition, the relative contents of AUR increase gradually in decomposing leaf litter of tree species, especially in temperate forests^7,8^.

Nevertheless, a suite of fungi is known to be capable of removing lignin and other recalcitrant compounds selectively from leaf tissues, resulting in whitening, or bleaching, of leaf litter. Such bleaching of leaf litter leads to the decrease in AUR content and the enhanced mass loss of leaf tissues and release of nitrogen compared with surrounding nonbleached portions^9^. These patterns of changes in bleached area and chemical composition have been investigated in decomposing leaf litter of tree species from temperate and tropical forests^10,11^, but the importance of the bleaching in decomposition processes on subtropical forest soil have rarely been evaluated quantitatively. Because lignin and other recalcitrant compounds were actively decomposed in subtropical forests^12,13^, we hypothesized that the bleaching could accelerate the decomposition of recalcitrant compounds and mobilization of nitrogen from leaf litter, contributing to the turnover of carbon and nutrients on subtropical forest soil.

The purposes of the present study were to elucidate the occurrence of bleached leaf area in decomposing leaf litter and to compare the composition of organic chemical components and organic and inorganic forms of nitrogen between bleached and nonbleached portions. As the appropriate study site, we chose a subtropical broad-leaved evergreen forest in southern Japan where bleached portions were recorded on the surface of leaf litter of at least 40 plant species in 20 plant families (Fig. S1, Table S1). Field incubation of leaf litter of six tree species (*Castanopsis sieboldii*, *Schima wallichii*, *Daphniphyllum teijsmannii*, *Persea thunbergii*, *Distylium racemosum*, and *Camellia japonica*) was performed over an 18-month period with the litterbag method to follow the expansion of bleached area and its chemical changes during decomposition. Bleached leaf litter of a total 20 tree species were then used for proximate analyses of organic chemical components, ^13^C solid-state nuclear magnetic resonance (NMR) analysis, and measurements of extractable organic and inorganic nitrogen so as to characterize chemical compositions of bleached leaf tissues, compared to adjacent nonbleached ones.

## Results

### Remaining mass and bleached area of whole leaf litter

**Figure 1:**
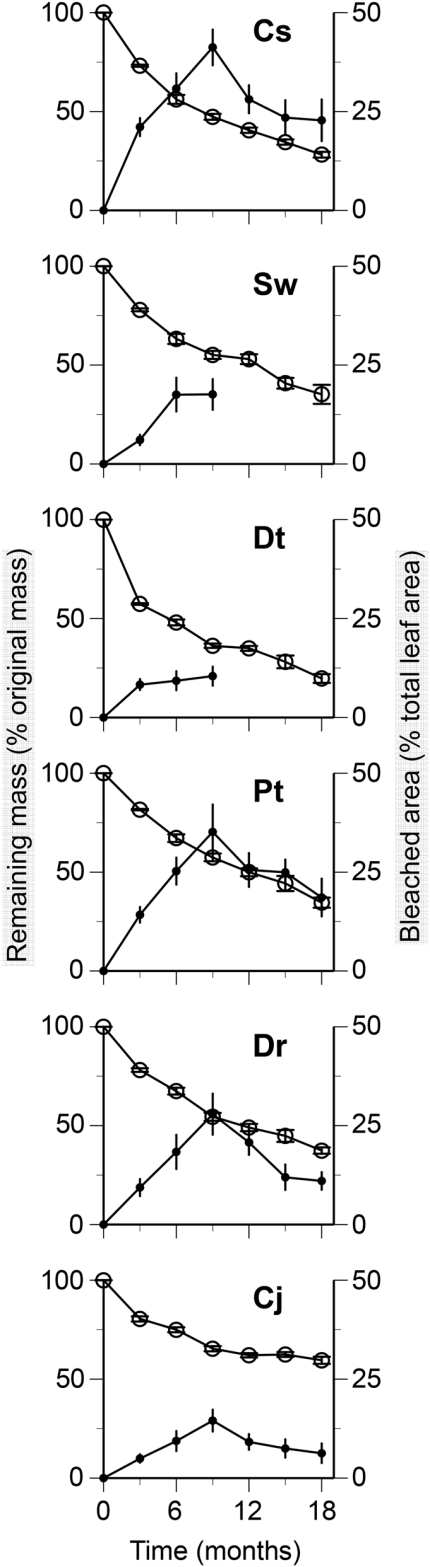
Changes in remaining mass (% original mass; left axis, open circle) and bleached area (% total leaf area; right axis, filled circle) during decomposition. Codes on the panels indicate the first letter of genus and species names of tree species. Bars indicate standard errors. No data were available for bleached area between 12 and 18 months in *Schima wallichii* and *Daphniphyllum teijsmannii* because of fragmentation of leaf litter.

At the end of 18 months of field incubation in litterbags, the remaining mass of leaf litter reached 20% (*Daphniphyllum teijsmannii*) to 60% (*Camellia japonica*) of the original mass (Fig. 1). In general, the decreases in remaining mass were relatively rapid during the first 9 months and then became slower thereafter. The bleached leaf area generally increased to reach 10% (*D. teijsmannii*) to 41% (*Castanopsis sieboldii*) of total leaf area at 9 months of decomposition; the proportion then decreased between 9 and 18 months (Fig. 1).

### Decomposition in bleached versus nonbleached portions

**Figure 2:**
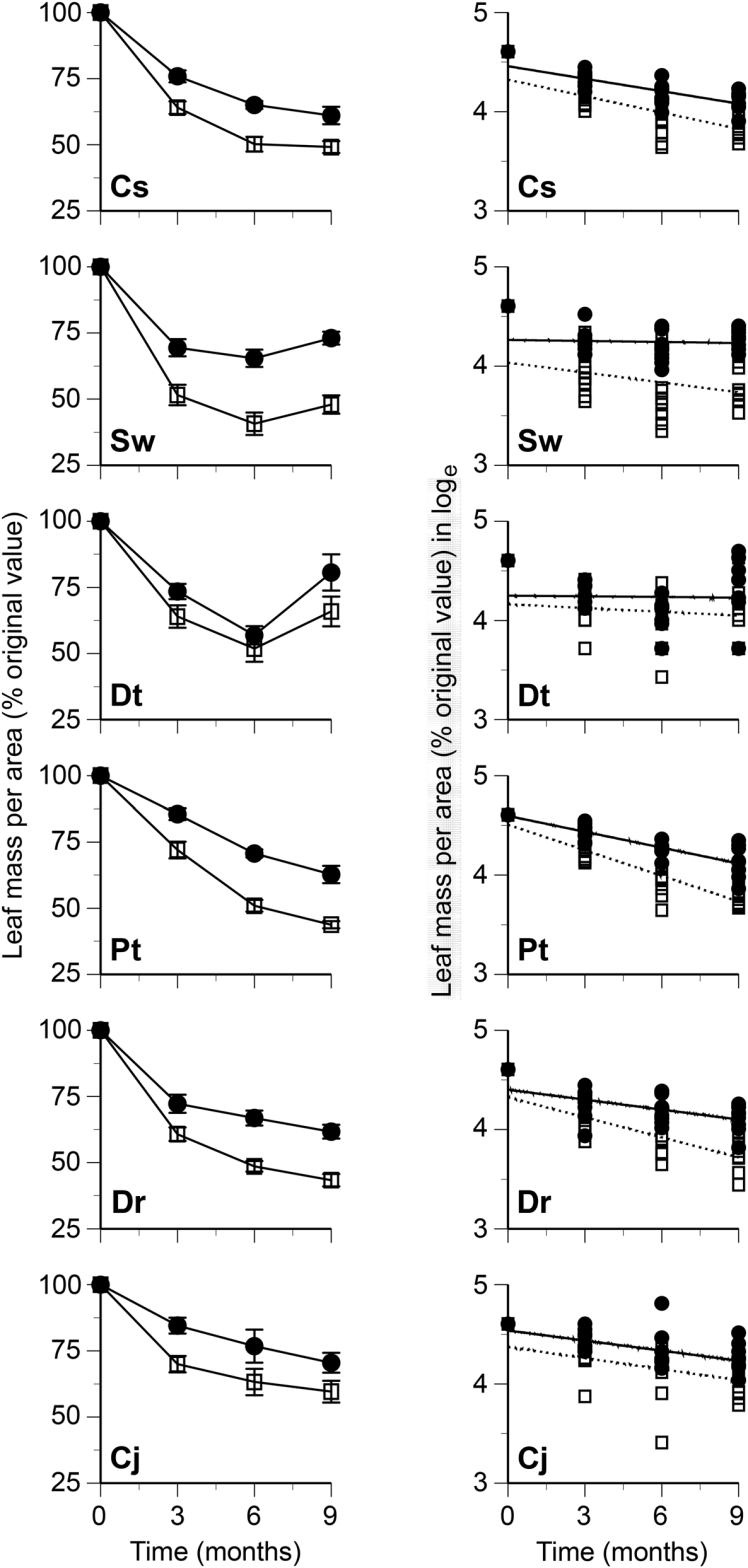
Changes in leaf mass per area (left) and relationship between time in months and remaining mass of leaf tissues (% original values of leaf mass per area) (right) in bleached (open square, dotted line) and nonbleached portions (filled circle, black line) during decomposition. Codes on the panels indicate the first letter of genus and species names of tree species. Bars indicate standard errors.

**Figure 3:**
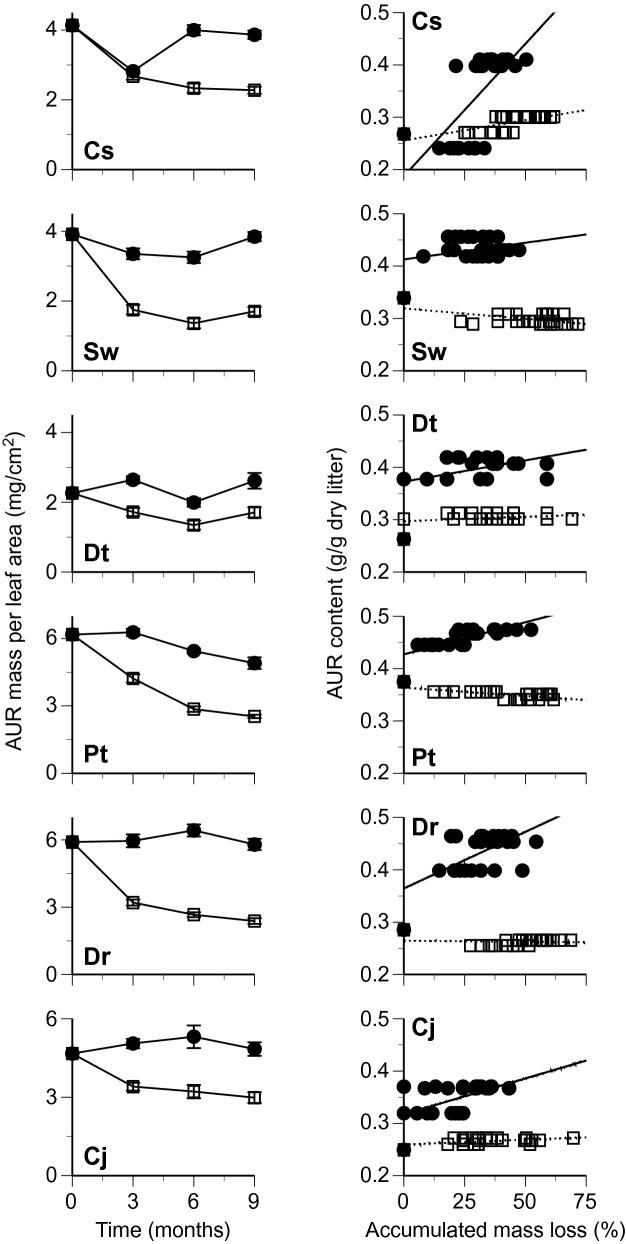
Changes in acid-unhyrolyzable residue (AUR) mass per leaf area (left) and relationship between accumulated mass loss and AUR content (right) in bleached (open square, dotted line) and nonbleached portions (filled circle, black line) during decomposition. Codes on the panels indicate the first letter of genus and species names of tree species. Bars indicate standard errors.

**Figure 4:**
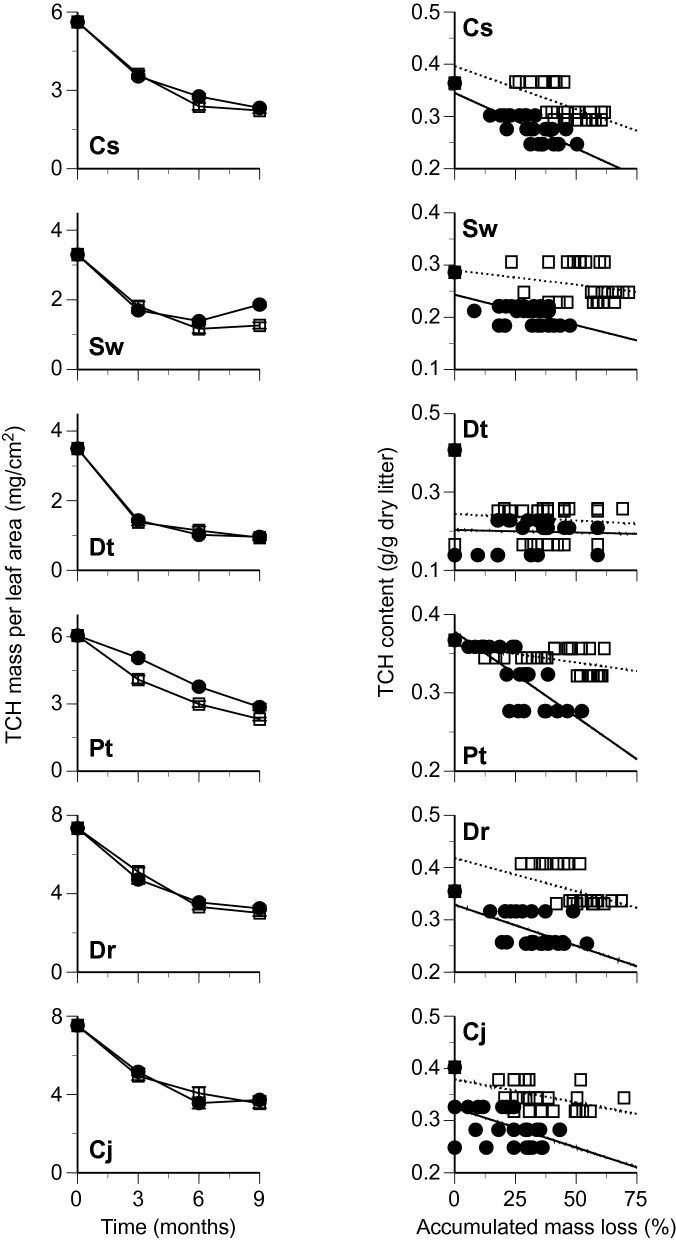
Changes in total carbohydrates (TCH) mass per leaf area (left) and relationship between accumulated mass loss and TCH content (right) in bleached (open square, dotted line) and nonbleached portions (filled circle, black line) during decomposition. Codes on the panels indicate the first letter of genus and species names of tree species. Bars indicate standard errors.

**Figure 5:**
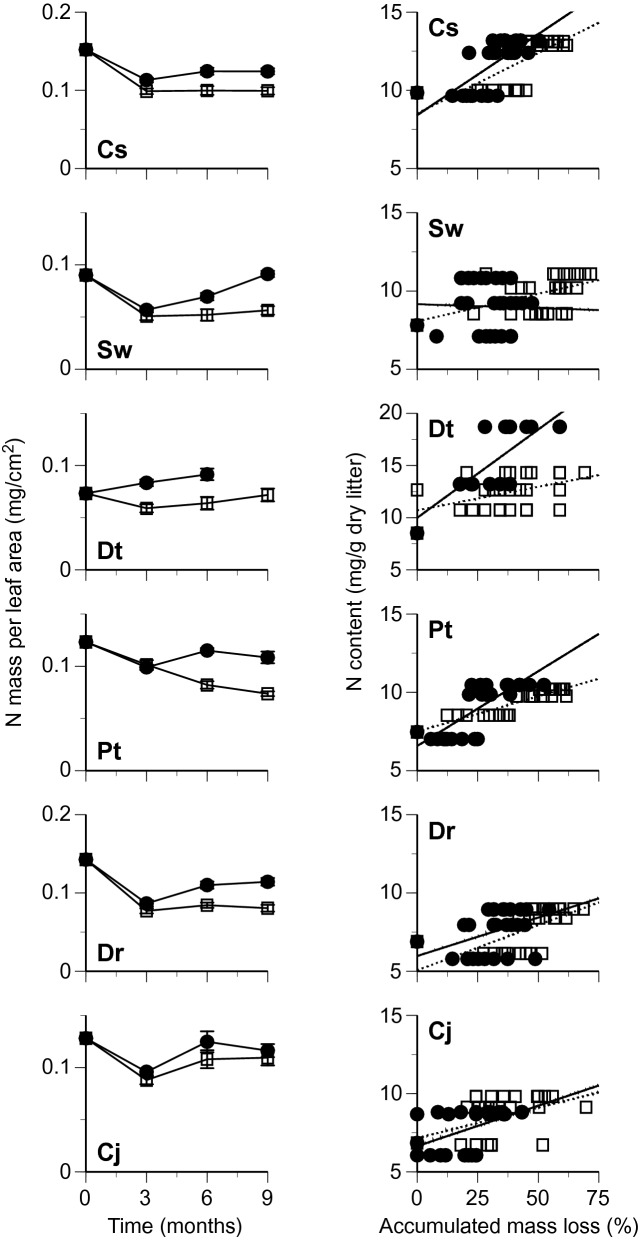
Changes in N mass per leaf area (left) and relationship between accumulated mass loss and N content (right) in bleached (open square, dotted line) and nonbleached portions (filled circle, black line) during decomposition. Codes on the panels indicate the first letter of genus and species names of tree species. Bars indicate standard errors. No data were available for total N content of the nonbleached portions of *D. teijsmannii* at 9 months of decomposition because of the small amount of sample.

Changes in leaf mass per area (LMA) and mass per leaf area of chemical components were followed separately for the bleached and nonbleached portions of leaf litter of six tree species during the first 9 months of decomposition. The LMA decreased during the decomposition, except for *S. wallichii* and *D. teijsmannii* at 9 months, and was generally lower in the bleached portions than in the nonbleached portions (Fig. 2). The AUR mass per area decreased slowly in the bleached portions, whereas in the nonbleached portions the AUR mass per area was relatively constant, or net increases of AUR mass occurred (Fig. 3). The changes in total carbohydrates (TCH) mass per area followed overall similar patterns between the bleached and nonbleached portions (Fig. 4). Total N mass per area decreased slowly or was relatively constant in the bleached portions, whereas it was generally greater in the nonbleached portions than in the bleached portions, and net increases of N mass occurred in some litter types (i.e., there was net N immobilization), especially in the nonbleached portions (Fig. 5).

Leaf mass per area (ln-transformed) in general decreased linearly with the duration of decomposition (Fig. 2). The regression equation between the LMA and decomposition time was statistically significant in eight out of the 12 cases (6 tree species × 2 portions) (Table 1). The mean value of the slopes for linear regression between the LMA and decomposition time was significantly lower in the bleached portions than in the nonbleached portions (Table 2). The AUR content was relatively constant in the bleached portions, whereas it increased in the nonbleached portions (Fig. 3). The regression equation between AUR content and accumulated mass loss was statistically significant in nine of the 12 cases (Table 1). The slopes for linear regression between AUR content and accumulated mass loss were significantly lower in the bleached portions than in the nonbleached portions (Table 2). The TCH content generally decreased in both the bleached and nonbleached portions (Fig. 4), and the regression equation was statistically significant in eight of the 12 cases (Table 1). The slopes for linear regression between TCH content and accumulated mass loss was not significantly different between the bleached portions and the nonbleached portions (Table 2). Total N content generally increased in both the bleached and nonbleached portions (Fig. 5), and the regression equation was statistically significant in 11 of the 12 cases (Table 1). The slopes for linear regression between N content and accumulated mass loss were not significantly different between the bleached portions and the nonbleached portions (Table 2).

**Table 1:** Slopes and intercepts of regression equations for linear relationships between leaf mass per area (LMA; % initial value, ln-transformed) and decomposition time in months and between contents of chemical components and accumulated mass loss (AML) in bleached (BL) and nonbleached (NB) portions of leaf litter.

	LMA vs time in months				AUR content vs AML			
	Slope	Intercept	R^2^	*P*	Slope	Intercept	R^2^	*P*
***Castanopsis sieboldii***								
BL	− 0.055	4.32	− 0.688	0.000	0.776	256	0.689	0.000
NB	− 0.042	4.46	− 0.755	0.000	5.056	187	0.682	0.000
***Schima wallichii***								
BL	− 0.033	4.03	− 0.314	0.104	− 0.394	319	− 0.546	0.003
NB	− 0.004	4.26	− 0.065	0.743	0.635	413	0.286	0.139
***Daphniphyllum teijsmannii***								
BL	− 0.013	4.16	− 0.122	0.535	0.162	297	0.289	0.135
NB	− 0.002	4.25	− 0.023	0.906	0.764	374	0.429	0.023
***Persea thunbergii***								
BL	− 0.086	4.51	− 0.881	0.000	− 0.321	364	− 0.653	0.000
NB	− 0.053	4.60	− 0.811	0.000	1.226	427	0.760	0.000
***Distylium racemosum***								
BL	− 0.067	4.33	− 0.772	0.000	− 0.040	265	− 0.083	0.674
NB	− 0.033	4.40	− 0.545	0.003	2.163	364	0.601	0.001
***Camellia japonica***								
BL	− 0.037	4.37	− 0.415	0.028	0.186	260	0.452	0.016
NB	− 0.034	4.54	− 0.512	0.005	0.921	328	0.448	0.017

	TCH content vs AML				Total N content vs AML			
	Slope	Intercept	R^2^	*P*	Slope	Intercept	R^2^	*P*
***Castanopsis sieboldii***								
BL	− 1.645	396	− 0.662	0.000	0.077	8.52	0.684	0.000
NB	− 2.122	345	− 0.802	0.000	0.104	8.41	0.709	0.000
***Schima wallichii***								
BL	− 0.552	290	− 0.260	0.182	0.035	8.06	0.484	0.009
NB	− 1.166	243	− 0.574	0.001	− 0.005	9.16	− 0.035	0.859
***Daphniphyllum teijsmannii***								
BL	− 0.300	243	− 0.094	0.633	0.044	10.77	0.450	0.016
NB	− 0.028	200	− 0.009	0.965	0.169	9.99	0.767	0.000
***Persea thunbergii***								
BL	− 0.438	361	− 0.461	0.013	0.045	7.49	0.905	0.000
NB	− 2.167	378	− 0.795	0.000	0.096	6.57	0.793	0.000
***Distylium racemosum***								
BL	− 1.268	418	− 0.491	0.008	0.058	5.06	0.630	0.000
NB	− 1.567	329	− 0.553	0.002	0.049	5.97	0.421	0.026
***Camellia japonica***								
BL	− 0.880	379	− 0.466	0.013	0.039	7.15	0.405	0.033
NB	− 0.953	311	− 0.362	0.058	0.035	7.06	0.396	0.037

**Table 2:** Chemical properties and decomposition in bleached (BL) and nonbleached (NB) portions of leaf litter.

Property	N	BL	NB	*t*-value
**Decomposition process**				
Slope of regression equation				
Leaf mass per area vs time in months	6	− 0.049 (0.011)	− 0.028 (0.008)	3.83*
AUR content vs accumulated mass loss	6	0.061 (0.173)	1.794 (0.690)	3.08*
TCH content vs accumulated mass loss	6	− 0.847 (0.214)	− 1.334 (0.329)	1.74ns
Total N content vs accumulated mass loss	6	0.050 (0.006)	0.075 (0.025)	1.04ns
**Chemical composition**				
Leaf mass per area (mg/cm^2^)	20	9.5 (0.7)	11.2 (0.7)	5.58***
Proximate organic chemical components (mg/g)				
AUR content	13	284 (12)	393 (15)	19.51***
TCH content	13	382 (18)	334 (11)	4.08**
EXT content	13	70(6)	68 (6)	0.64ns
Relative area of ^13^C NMR spectra (%)				
Alkyl-C	12	22(2)	24(2)	1.59ns
O-alkyl-C	12	63 (1)	60 (1)	3.06*
Aromatic-C	12	12 (1)	13 (1)	0.92ns
Carbonyl-C	12	2.8 (0.2)	3.3 (0.2)	3.11**
Dissolved nitrogen (μN/g)				
Total extractable nitrogen (TEN)	13	565 (57)	313 (22)	5.98***
Extractable organic nitrogen (EON)	13	451 (57)	227 (21)	5.46***
NH^+^_4_-N	13	101 (13)	75 (9)	3.35**
NO_3_^–^-N	13	9.6 (1.8)	8.8 (1.6)	0.84ns
NO_2_^–^-N	13	3.4 (0.5)	2.9 (0.4)	1.54ns
%EON	13	78 (3)	71 (3)	5.27***

N, number of tree species examined. Values are means with standard errors in parentheses.

Paired *t*-test, ***P < 0.001, **P < 0.01, *P < 0.05, ns non-significant.

### Chemical composition in bleached and nonbleached portions

The mean values of LMA and AUR content were significantly lower in bleached than in nonbleached portions for leaf litter of 20 and 12 tree species, respectively (Table 2). In contrast, the mean value of TCH content was significantly higher in bleached than in nonbleached portions for leaf litter (Table 2). The mean value of EXT content was not significantly different between bleached and nonbleached portions (Table 2).

O-alkyl-C was the predominant component, accounting for 51.1–70.7% of the NMR spectra of leaves of 12 tree species, followed by alkyl-C, aromatic-C, and carbonyl-C (Fig. S2, Table S3). The mean relative area of the signal for O-alkyl-C was significantly higher, whereas that for carbonyl-C was significantly lower, in bleached portions than in nonbleached portions of leaf litter (Table 2). The relative area of the signal for alkyl-C and aromatic-C was not significantly different between the bleached portions and the nonbleached portions (Table 2).

Total extractable nitrogen (TEN) ranged from 154 to 1028 µg N/g (Table S4), and its mean value was significantly higher in bleached portions than in nonbleached portions for leaf litter of 13 tree species (Table 2). The content of extractable organic nitrogen (EON) ranged from 128 to 922 µgN/g, accounting for 56.9–92.2% of TEN (Table S4), and mean values of EON and its proportion with respect to TEN (%EON) were significantly greater in bleached than in nonbleached portions (Table 2). NH_4_^+^-N accounted for 69–97% of the three forms of inorganic nitrogen (NH_4_^+^-N, NO_3_^–^-N, and NO_2_^–^-N) (Table S4). The content of NH_4_^+^-N was significantly greater in bleached than in nonbleached portions, whereas the contents of NO_3_^–^-N and NO_2_^–^-N were not significantly different between bleached and nonbleached portions (Table 2).

## Discussion

The decomposition processes of leaf litter of six tree species were divided into two stages, which corresponded to the increase and decrease of bleached leaf area (Fig. 1): the first 9 month-period was characterized by the relatively rapid mass loss of whole leaf litter and concomitant increase of bleached leaf area, whereas the mass loss was slowed down in accordance with the decreased bleached leaf area from 9 to 18 months. This two-stage pattern was consistent with previous reports that followed the patterns of change in bleached leaf area during decomposition. For example, the bleached area on leaf litter of *Camellia japonica* increased rapidly up to 17% of total area during the first 2 months and then decreased thereafter over an 18-month period in a temperate forest^10^. The bleached area on *Shorea obtusa* leaf litter increased linearly with time to reach 30% of total leaf area at 9 months of decomposition in a tropical forest^11^. The present study agrees with these previous ones explicitly showing that the expansion of bleached area contributed to the faster decomposition of whole leaf litter in the initial stage. Two explanations may account for the decrease of bleached area in the later stage. First, some bleached portions could become darkened due to successive decomposition of delignified carbohydrates^14^. Secondly, leaf tissues in the bleached portions could be fragmented and lost faster than those in nonbleached portions, resulting in an apparent increase of nonbleached area with respect to the remaining total leaf area. Such fragmentation may also account for the increase of LMA in *S. wallichii* and *D. teijsmannii* at 9 months that was possibly due to the loss of leaf lamina and the persistence of leaf vein that contributed more to LMA than lamina.

The consistently lower values of LMA and AUR content in bleached portions than in adjacent nonbleached portions of leaf litter (Figs. 2 and 3) indicate the acceleration of mass loss of leaf tissue in bleached portions by the selective decomposition of recalcitrant compounds registered as AUR. The slope for linear regression between AUR content and accumulated mass loss is an index representing the degree of selective decomposition of AUR^15^ which was significantly lower in bleached than in nonbleached portions (Table 2), demonstrating the more selective decomposition of recalcitrant compounds in the bleached portions. The relative area of carbonyl-C in ^13^C NMR spectra was lower in the bleached than in nonbleached portions (Table 2), indicative of the loss of carbonyl carbons of lignin, and carboxylic-C in tannins^16^. Such selective decomposition of these recalcitrant compounds led to relative increase of total carbohydrates and O-alkyl-C in the bleached portions (Table 2). The selective loss of lignin in bleached leaf litter is typical of decomposition processes in tropical forests^17^, whereas the selective decomposition of carbohydrates found in the nonbleached portions is commonly observed during decomposition in temperate forests^18^. The net increase of AUR mass per area in the nonbleached portions of some leaf litter is possibly due to the formation of secondary substances registered as AUR during decomposition^1,2^.

The selective decomposition of recalcitrant compounds was associated with the greater pool sizes of total extractable N (TEN) attached to the bleached portions (Table 2), which were attributed to enhanced leaching of low molecular weight compounds and concomitant mineralization of organic N^19^. The increased pool size of TEN in the bleached portions was mainly attributed to the increase of extractable organic N (EON) and NH_4_^+^-N, with a greater contribution of EON (as an increase of %EON in the bleached portions, Table 2). The increase of NH_4_^+^-N was similar to the enhanced N mineralization in bleached humus produced in temperate forests by the activity of fungi to cause selective decomposition of lignin and other recalcitrant compounds^20^. Previous studies of leaf litter decomposition already showed that N mobilization from litter is closely associated with AUR decomposition^21,22^.

The present study demonstrated that the expansion of bleached area plays a dominant role in the transformation and turnover of organic compounds and N in decomposing leaf litter of the study site. Studying decomposition processes and chemical changes in bleached portions and comparing with those in nonbleached portions are promising in elucidating the lignin control on the decomposition. This is especially true in tropical and subtropical forests where the occurrence of bleached area in decomposing leaf litter is a common phenomenon on the forest floor^11,23^. Moreover, bleached leaf litter are suitable for relating the functional roles of ligninolytic fungi to the decomposition processes. In fact, fieldwork conducted at the same study site documented a diverse suite of fungi associated with the bleaching of leaf litter^24,25^. The localized colonization and bleaching by these fungi yield the small-scale heterogeneity of decomposition of recalcitrant compounds and N mobilization within the single leaf litter. In this respect, we should note a limitation of the present study that data are lacking regarding micro-arthropods, in spite of the use of litterbags with 2 mm-mesh size that can allow access to leaf litter by such mesofauna as collembola and acari^26^. It is possible that parts of recalcitrant tissues in bleached portions of leaf litter be fragmented by soil fauna to be incorporated into soil underneath and processed further by decomposer organisms^27^. Finally, we used leaf litter of six tree species as materials, but the occurrence of such bleached area was encountered on leaf litter of at least 40 tree species in the subtropical forest in the study site (Table S1). A recent study also elucidated that the occurrence of bleached area on leaf litter could vary with climates^23^. Further studies are needed to explore the importance of bleaching processes in the decomposition of diverse leaf litter in other subtropical forests and its variability between climatic regions.

## Materials and methods

### Study site

The present study was conducted in evergreen broad-leaved subtropical forests in the northern part of Okinawa Island, south-western Japan. Samples were collected in a secondary forest within Yona Experimental Forest of University of the Ryukyus (26°9′ N, 128°5′ E, ca 250–330 m a.s.l.). The mean annual temperature was 20.7 °C and the annual precipitation was 2487 mm. The topography is hilly and dissected. The bedrock is composed of sandstone and slate, and yellow soil has developed. The forest stand was dominated by *Castanopsis sieboldii* and *Schima wallichii* ssp. *liuliuensis* with a maximum height of 20 m^28^.

### Litterbag experiment

Decomposition of leaf litter of six tree species (*C. sieboldii*, *S. wallichii*, *Daphniphyllum teijsmannii*, *Persea thunbergii*, *Distylium racemosum*, and *Camellia japonica*) was studied using a litterbag method, according to the procedure detailed previously^13^. These six tree species are dominant components of the forest canopy in the study site^28^. In short, a study plot of 50 m × 10 m (500 m^2^) was laid out in Yona Experimental Forest and was divided into 125 grids of 2 × 2 m. Freshly fallen leaves of six tree species were collected from the soil surface in March 2008. The leaves were dried in an oven at 40 °C for 1 week to a constant mass. Leaf litter (4.00 g) was placed in litterbags (24 × 18 cm) made of nylon with a mesh size of approximately 2 mm and incubated within the 500 m^2^ study plot for 18 months from April 2008 to October 2009. Nine litterbags per tree species were retrieved at 3, 6, 9, 12, 15, and 18 months after initiation of the experiment and used for measurement of the remaining mass of whole leaf litter^13^. In the present study, the bleached leaf area and leaf mass per area (LMA) and chemical compositions of bleached and nonbleached portions were then measured as described below. The LMA indicates the remaining mass of leaf tissues and represents the extent of decomposition in the bleached and nonbleached portions. Leaf litters of *S. wallichii* and *D. teijsmannii* collected at 12, 15 and 18 months of decomposition were too fragmented to measure bleached leaf area.

### Measurement and chemical analyses

Leaves were pressed between layers of plywood and paper and oven-dried at 40 °C for 1 week. The leaves were photocopied, scanned, and measured for the total leaf area and the proportion of bleached area according to the method described previously^29^. A 6-mm-diameter cork borer was then used to excise leaf disks, avoiding the primary vein, from the bleached area and surrounding nonbleached area of the same leaves collected for the first 9 months of decomposition. The disks were oven-dried again at 40 °C for 1 week and weighed to calculate LMA. The disks were combined to make 1 sample each of bleached and nonbleached leaf area for each tree species collected at each sampling occasion and used for chemical analyses as described below. Leaf disks could not be excised from leaves collected at 12, 15, and 18 months of decomposition because of fragmentation.

Litter materials were ground in a laboratory mill (0.5-mm screen). The amount of acid unhydrolyzable residue (AUR) and total carbohydrates (TCH) was estimated by means of gravimetry as acid-insoluble residue, using hot sulfuric acid digestion^30^ and by a phenol–sulfuric acid method^31^. Total N content was measured by automatic gas chromatography (NC analyzer SUMIGRAPH NC-900, Sumitomo Chemical Co., Osaka, Japan). Details of the methods followed Osono^13^. The contents of AUR and TCH were expressed in g/g dry litter, and that of total N was in mg/g dry litter. The mass of these components per leaf area was calculated by multiplying the contents and LMA. The AUR fraction contains a mixture of organic compounds in various proportions, including condensed tannins, phenolic and carboxylic compounds, alkyl compounds such as cutins, and true lignin^16^. No data were available for total N content of the nonbleached portions of *D. teijsmannii* at 9 months of decomposition because of the small amount of sample.

To analyze the chemical composition of bleached leaf tissues more in detail and to compare it with that of nonbleached portions for multiple tree species, samples of bleached leaf litter were collected during fieldworks in March 2007 and in April 2011. These bleached leaf litter were separated into bleached and nonbleached litter samples to be used for measurement and chemical analyses (Table S1). Bleached leaf litter of 20 tree species was used for measurement of LMA, and the samples of 13 of the 20 tree species were further analyzed for the contents of AUR and TCH, as described above (Table S2). Samples were extracted with alcohol-benzene at room temperature (15–20 °C) to remove extractives (EXT; soluble polyphenols, hydrocarbons, and pigments) and to calculate the content of this fraction.

Solid-state Cross polarization (CP) magic angle spinning (MAS) ^13^C NMR spectra of bleached and nonbleached litter samples for 12 tree species were obtained with an Alpha 300 FT NMR system (JEOL, Tokyo) operating at 75.45 MHz under the following conditions^32^: pulse repetition time of 3.1 s, CP contact time of 1 ms, sweep width of 35 kHz, acquisition time of 0.117 s, and MAS of 6 kHz. The finely powdered sample was tightly packed into a high-speed spinning NMR tube (rotor: zirconia, cap: KEL-F, 6-mm i.d., JEOL). Chemical shifts are quoted with respect to tetramethylsilane but were determined by referring to an external sample of adamantane (29.50 ppm). The ^13^C NMR spectra (Fig. S2) were divided into four chemical shift ranges, as follows: 0 to 45 ppm for alkyl-C (including major C of cutins and suberins), 45 to 110 ppm for O-alkyl-C (oxygen-substituted C in alcohols and ethers, including cellulose, hemicellulose, and other polysaccharides), 110 to 160 ppm for aromatic C (including mainly condensed tannins, hydrolyzable tannins, and lignin), and 160 to 190 ppm for carbonyl C (including carboxylic-C and carbonyl-C)^33^. The relative area of these chemical shift regions was calculated for each spectrum as the percentage of total area by using computer software ALICE 2 for Windows (JEOL) (Table S3).

Nitrogen attached to leaf litter was determined by extraction and colorimetric analyses of the extractants for 13 tree species. Approximately 100 mg of bleached or nonbleached leaf litter was shaken with 10 ml of 2 M KCl in a 15-ml centrifuge tube on a shaker for 1 h. The suspension was centrifuged at 3000 rpm for 10 min and filtered with glass fiber filters (GF/F, Whatman). The total extractable nitrogen (TEN) in the extractants was measured by the alkali persulfate digestion method^34^. Ammonium-nitrogen (NH_4_^+^-N), nitrate-nitrogen (NO_3_^–^-N), and nitrite-nitrogen (NO_2_^–^-N) were determined colorimetrically^35^ for the pre-digested samples. Extractable organic nitrogen (EON) was calculated subtracting these three forms of inorganic nitrogen from TEN (Table S4).

### Statistical analyses

Linear relationships between LMA and decomposition time and between contents of AUR, TCH, and total N and accumulated mass loss of leaf tissue were examined separately for bleached and nonbleached portions according to the following equations:

1 $${\text{Ln}}\,\left[ {{\text{LMA}}\,\left( {\% \,{\text{original}}\,{\text{value}}} \right)} \right] \, = a + b \times \, \left( {{\text{time}}\,{\text{in}}\,{\text{months}}} \right)$$

2 $${\text{AUR}},{\text{TCH}},{\text{and}}\,{\text{N}}\,{\text{content }} = a + b \times \left( {{\text{accumulated}}\,{\text{mass}}\,{\text{loss}}\,{\text{of}}\,{\text{leaf}}\,{\text{tissue}}} \right)$$

Accumulated mass loss of leaf tissue of bleached and nonbleached portions after a given period was calculated as the loss of LMA relative to the initial LMA values, expressed as a percentage. Intercepts (*a*) and slopes (*b*) of regression equations were calculated for the linear relationships using least-square regression^15^. The slope of the regression Eq. (1) represented the decomposition constant^36^. The slopes of the regression Eq. (2) describing AUR and N dynamics represented the N concentration increase rate and the lignin concentration increase rate, respectively^15^. A paired *t*-test was used to evaluate the difference between bleached and nonbleached portions in the slopes of regression equations for LMA, AUR, TCH, and N in decomposing leaf litter of 6 tree species and in LMA, contents of proximate organic chemical components, relative area of ^13^C NMR spectra, and contents of dissolved N in leaf litter of multiple tree species.

## Supplementary Information

Supplementary Information.

## References

[1] Berg B, McClaugherty C (2003). Plant Litter, Decomposition, Humus Formation, Carbon Sequestration.

[2] Berg B, Laskowski R (2006). Litter decomposition: a guide to carbon and nutrient turnover. Adv. Ecol. Res..

[3] Coûteaux MM, Bottner P, Berg B (1995). Litter decomposition, climate and litter quality. Trends Ecol. Evol..

[4] Aerts A (1997). Climate, leaf litter chemistry and leaf litter decomposition in terrestrial ecosystems: a triangular relationship. Oikos.

[5] Hou PCL, Zou X, Huang CY, Chien HJ (2005). Plant litter decomposition influenced by soil animals and disturbance in a subtropical rainforest of Taiwan. Pedobiologia.

[6] Hättenschwiler S, Coq S, Barantal S, Handa IT (2011). Leaf traits and decomposition in tropical rainforests: revisiting some commonly held views and towards a new hypothesis. New Phytol..

[7] Berg B, Ekbohm G, McClaugherty C (1984). Lignin and holocellulose relations during long-term decomposition of some forest litters. Long-term decomposition in a Scots pine forest. IV. Can. J. Bot..

[8] Aber JD, Mellilo JM, McClaugherty CA (1990). Predicting long-term patterns of mass loss, nitrogen dynamics, and soil organic matter formation from initial fine litter chemistry in temperate forest ecosystems. Can. J. Bot..

[9] Osono T (2020). Functional diversity of ligninolytic fungi associated with leaf litter decomposition. Ecol. Res..

[10] Koide K, Osono T, Takeda H (2005). Fungal succession and decomposition of *Camellia japonica* leaf litter. Ecol. Res..

[11] Osono T, Ishii Y, Takeda H, Seramethakun T, Khamyong S, To-Anun C, Hirose D, Tokumasu S, Kakishima M (2009). Fungal succession and lignin decomposition on *Shorea obtusa* leaves in a tropical seasonal forest in northern Thailand. Fungal Divers..

[12] Ono K, Hiradate S, Morita S, Hirai K (2013). Fate of organic carbon during decomposition of different litter types in Japan. Biogeochemistry.

[13] Osono T (2017). Leaf litter decomposition of 12 tree species in a subtropical forest in Japan. Ecol. Res..

[14] Osono T, Hirose D (2009). Effects of prior decomposition of *Camellia japonica* leaf litter by an endophytic fungus on the subsequent decomposition by fungal colonizers. Mycoscience.

[15] Berg, B., McClaugherty, C. & Johansson, M.B. *Chemical Changes in Decomposing Litter Can Be Systemized with Respect to the Initial Chemical Composition of the Litter* (Swedish University of Agricultural Sciences report 74, 1997).

[16] Preston CM, Trofymow JA, Sayer BG, Niu J (1997). ^13^C nuclear magnetic resonance spectroscopy with cross-polarization and magic-angle spinning investigation of the proximate-analysis fractions used to assess litter quality in decomposition studies. Can. J. Bot..

[17] Hirobe M, Sabang J, Bhatta BK, Takeda H (2004). Leaf-litter decomposition of 15 tree species in a lowland tropical rain forest in Sarawak, dynamics of carbon, nutrients, and organic constituents. J. For. Res..

[18] McTiernan KB (2003). Changes in chemical composition of *Pinus sylvestris* needle litter during decomposition along a European coniferous forest climatic transect. Soil Biol. Biochem..

[19] Hobara S, Koba K, Osono T, Tokuchi N, Ishida A, Kameda K (2005). Nitrogen and phosphorus enrichment and balance in forests colonized by cormorants: implications of the influence of soil adsorption. Plant Soil.

[20] Hintikka V (1970). Studies on white-rot humus formed by higher fungi in forest soils. Comm. Inst. For. Fenn..

[21] Berg B, McClaugherty C (1989). Nitrogen and phosphorus release from decomposing litter in relation to the disappearance of lignin. Can. J. Bot..

[22] Kuyper TW, Bokeloh DJ (1994). Ligninolysis and nitrification in vitro by a nitrotolerant and a nitrophobic decomposer basidiomycetes. Oikos.

[23] Osono T, Matsuoka S, Hirose D (2020). Diversity and geographic distribution of ligninolytic fungi associated with *Castanopsis sieboldii* leaf litter in Japan. Front. Microbiol..

[24] Osono T, Ishii Y, Hirose D (2008). Fungal colonization and decomposition of *Castanopsis sieboldii* leaf litter in a subtropical forest. Ecol. Res..

[25] Hirose D, Matsuoka S, Osono T (2013). Assessment of the fungal diversity and succession of ligninolytic endophytes in *Camellia japonica* leaves using clone library analysis. Mycologia.

[26] Alhamd L, Arakaki S, Hagihara A (2004). Decomposition of leaf litter of four tree species in a subtropical evergreen broad-leaved forest, Okinawa Island, Japan. For. Ecol. Manag..

[27] Uchida T, Kenako N, Ito MT, Futagami K, Sasaki T, Sugimoto A (2004). Analysis of the feeding ecology of earthworms (Megascolecidae) in Japanese forests using gut content fractionation and δ^15^N and δ^13^C stable isotope natural abundances. Appl. Soil Ecol..

[28] Enoki T (2003). Microtopography and distribution of canopy trees in a subtropical evergreen broad-leaved forest in the northern part of Okinawa Island, Japan. Ecol. Res..

[29] Hagiwara Y, Matsuoka S, Hobara S, Mori AS, Hirose D, Osono T (2015). Bleaching of leaf litter and associated fungi in subboreal and subalpine forests. Can. J. Microbiol..

[30] King HGC, Heath GW (1967). The chemical analysis of small samples of leaf material and the relationship between the disappearance and composition of leaves. Pedobiologia.

[31] Dubois M, Gilles KA, Hamilton JK, Rebers PA, Smith F (1956). Colorimetric method for determination of sugars and related substances. Anal. Chem..

[32] Hiradate S, Hirai H, Hashimoto H (2006). Characterization of allophanic Andisols by solid-state ^13^C, ^27^Al, and ^29^Si NMR and by C stable isotopic ratio, δ^13^C. Geoderma.

[33] Osono T, Azuma JI, Hirose D (2014). Plant species effect on the decomposition and chemical changes of leaf litter in grassland and pine and oak forest soils. Plant Soil.

[34] Sollins P, Glassmann C, Paul EA, Swanston C, Lajtha K, Heil JW, Elliott ET, Robertson GP (1999). Soil carbon and nitrogen—pools and fractions. Standard Soil Methods for Long-Term Ecological Research.

[35] Mulvaney RL, Sparks DL (1996). Nitrogen—inorganic form. Methods of Soil Analysis, Part 3, Chemical Methods. SSSA Book Series no. 5.

[36] Olson J (1963). Energy storage and the balance of produces and decomposers in ecological systems. Ecology.

