# Supplementary material for: Bleaching of leaf litter accelerates the decomposition of recalcitrant components and mobilization of nitrogen in a subtropical forest
Source: Sci Rep. 2021 Jan 19;11:1787. doi: 10.1038/s41598-021-81206-7 (PMC7815825; doi:10.1038/s41598-021-81206-7)
Supplement: Supplementary file 1 — Supplementary Information. [file 41598_2021_81206_MOESM1_ESM.pdf]

Electronic Supplementary Material

Bleaching of leaf litter accelerates the decomposition of recalcitrant components and mobilization of nitrogen in a subtropical forest

Takashi Osono, Syuntaro Hiradate, Satoru Hobara

Fig. S1. Images of selected bleached leaf litter in a subtropical forest, Okinawa, southern Japan. (a) *Castanopsis sieboldii* , (b) *Schima wallichii* ,(c) *Persea thunbergii* , (d) *Distylium racemosum* , (e) *Camellia japonica* , (f) *Camellia sasanqua* , (g) *Elaeocarpus japonicus* , (h) *Tricalysia dubia* , (i) *Cinnamomum doederleinii* , (j) *Rhododendron tashiroi* , (k) *Antidesma rigida* , (l) *Syzygium buxifolium* , (m) *Dendropanax trifidus* , (n) *Meliosma squamulata* , (o) *Podocarpus nagi* , (p) *Quercus miyagii* . Bar = 10 mm.

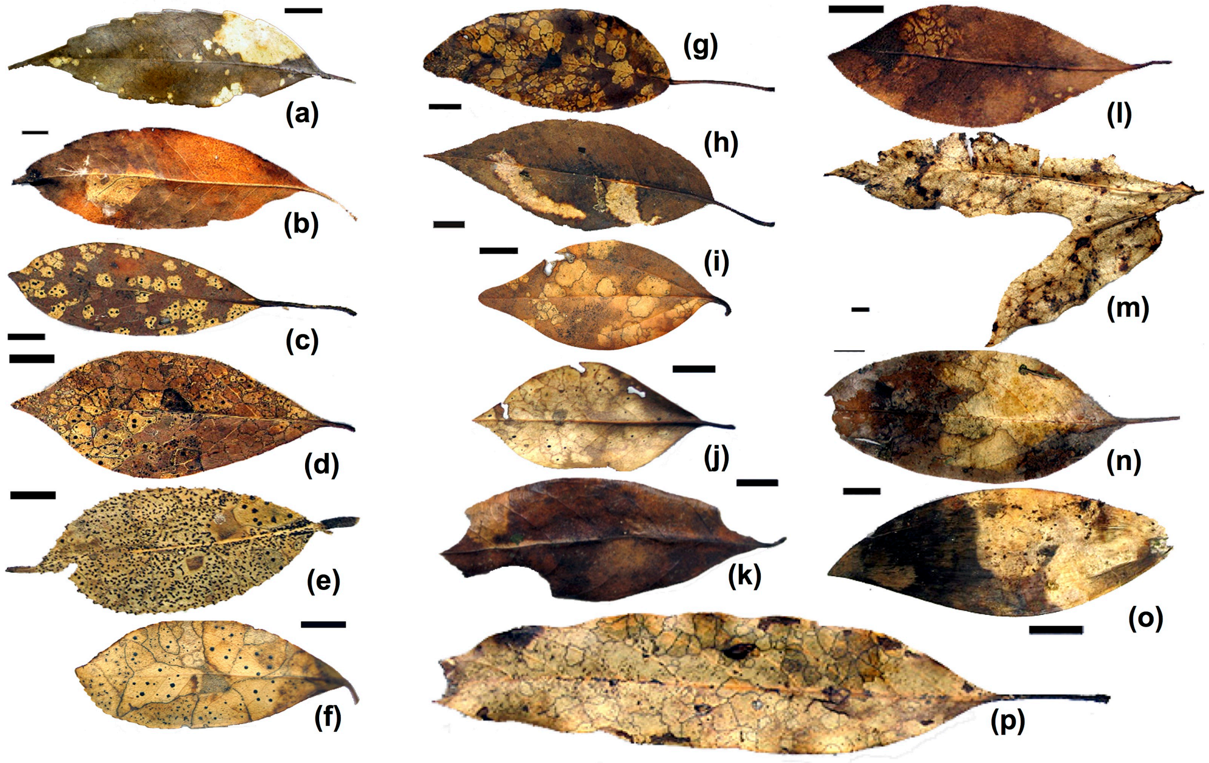

Table S1. Plant species of which bleached portions were observed on leaf litter, the frequency of occurrence of bleached litter, and plant species examined in the present study.

Plant species[1]	Plant family	Extent of bleached portions[2]	Frequency of occurrence (%)[3]	LMA	Proximate analysis	13C NMR	Dissolved N	Litterbag
Ilex goshiensis	Aquifoliaceae	4	0					
Ilex liukiuensis	Aquifoliaceae	3	0					
Ilex maximowicziana var. *kanehirae*	Aquifoliaceae	5	22	1	1	1	1	
Dendropanax trifidus	Araliaceae	4	19	1	1			
Schefflera octophylla	Araliaceae	1	0					
Daphniphyllum teijsmannii	Daphniphyllaceae	2	15	1				1
Elaeocarpus japonicus	Elaeocarpaceae	4	14	1	1	1	1	
Elaeocarpus sylvestris	Elaeocarpaceae	3	0					
Rhododendron tashiroi	Ericaceae	5	2	1		1	1	
Vaccinium wrightii	Ericaceae	2	2					
Antidesma rigida	Euphorbiaceae	1	0					
Sapium japonicum	Euphorbiaceae	2	0					
Castanopsis sieboldii	Fagaceae	5	98	1	1	1	1	1
Quercus miyagii	Fagaceae	5	2	1			1	
Distylium racemosum	Hamamelidaceae	5	60	1	1	1	1	1
Cinnamomum doederleinii	Lauraceae	3	11	1				
Neolitsea aciculata	Lauraceae	2	1					
Neolitsea sericea	Lauraceae	3	12	1	1		1	
Persea japonica	Lauraceae	5	0					
Persea thunbergii	Lauraceae	5	42	1	1	1		1
Mucuna macrocarpa	Leguminosae	1	1					
Heterosmilax japonica	Liliaceae	3	1					
Myrica rubra	Myricaceae	4	3	1	1	1	1	
Ardisia quinquegona	Myrsinaceae	3	1					
Myrsine seguinii	Myrsinaceae	4	22	1	1	1	1	
Syzygium buxifolium	Myrtaceae	3	9	1				
Pinus luchuensis	Pinaceae	2	0					
Podocarpus nagi	Podocarpaceae	4	3	1	1	1	1	
Lasianthus curtisii	Rubiaceae	1	0					
Psychotria rubra	Rubiaceae	2	0					
Randia canthioides	Rubiaceae	1	0					
Tricalysia dubia	Rubiaceae	2	0					
Meliosma squamulata	Sabiaceae	3	6	1	1	1	1	
Symplocos okinawaensis	Symplocaceae	5	0					
Camellia japonica	Theaceae	5	20	1	1	1	1	1
Camellia lutchuensis	Theaceae	4	0					
Camellia sasanqua	Theaceae	5	5	1				
Schima wallichii	Theaceae	3	36	1	1	1	1	1
Ternstroemia gymnanthera	Theaceae	5	6	1				
Tutcheria virgata	Theaceae	2	0					
Number of plant family		20						
Number of plant genera		33						
Number of plant species		40		20	13	12	13	6

[1] Plant taxonomy follows Angiosperm Phylogeny Group (2009). Scientific names of tree species follow Satake et al. (1989).

[2] Extent of bleached portions within single leaves was scored visually into five classes: 1, bleached in 1% to 20% of total leaf area; 2, bleached in 21% to 40%; 3, bleached in 41% to 60%; 4, bleached in 61% to 80%; and 5, bleached in 81% to 100%. The maximal score recorded during the study period is given in the table.

[3] Frequency of occurrence denotes the percentage of the number of grids within which the bleached litter and/or associated fruiting bodies were observed with respect to the total number of grids examined (125).

References

Angiosperm Phylogeny Group, 2009. An update of the Angiosperm Phylogeny Group classification for the orders and families of flowering plants: APG III. Botanical Journal of the Linnean Society 161, 105-121.

Satake, Y., Hara, H., Watari, S., Tominari, T., 1989. Wild Flowers of Japan. Woody Plants. Heibonsha, Tokyo (in Japanese).

Table S2. Leaf mass per area (mg/cm^2), contents (mg/g) of organic chemical components [acid unhydrolyzable residue (AUR), total carbohydrates (TCH), and extractives (EXT)] by proximate analysis, and lignocellulose index (LCI) of bleached (BL) and nonbleached portions (NB) of leaf litter of 20 plant species. nd, no data. LCI = TCH content / (TCH content + AUR content).

Plant species	Leaf mass per area					Proximate analysis							
	BL		NB		N[1]	AUR		TCH		LCI		EXT	
	Mean	se	Mean	se		BL	NB	BL	NB	BL	NB	BL	NB
Castanopsis sieboldii	8.6	0.5	10.5	0.6	5	258	370	357	300	0.581	0.448	79	70
Distylium racemosum	12.3	0.6	14.3	0.6	5	224	353	427	370	0.656	0.512	49	60
Persea thunbergii	9.8	0.7	12.5	0.6	5	337	446	359	368	0.516	0.452	62	50
Schima wallichii	6.9	0.2	8.7	0.3	5	318	450	348	271	0.523	0.376	83	73
Myrsine seguinii	7.5	0.2	8.7	0.2	4	335	473	325	281	0.492	0.373	67	71
Ilex maximowicziana var. *kanehirae*	10.0		11.4		2	230	311	362	349	0.611	0.528	108	89
Camellia japonica	14.5	1.2	17.3	1.3	5	250	344	436	338	0.635	0.496	69	62
Dendropanax trifidus	7.6	0.5	7.2	0.3	4	244	312	318	302	0.565	0.492	103	121
Daphniphyllum teijsmannii	7.5		10.3		2	nd	nd	nd	nd	nd	nd	nd	nd
Elaeocarpus japonicus	6.8	0.4	7.8	0.6	5	348	454	347	325	0.499	0.417	50	56
Neolitsea sericea	7.4	0.4	8.9	0.5	4	317	447	314	313	0.498	0.412	92	92
Cinnamomum doederleinii	12.6		12.0		1	nd	nd	nd	nd	nd	nd	nd	nd
Syzygium buxifolium	5.7		9.2		1	nd	nd	nd	nd	nd	nd	nd	nd
Ternstroemia gymnanthera	14.5		15.6		1	nd	nd	nd	nd	nd	nd	nd	nd
Meliosma squamulata	7.9		8.6		2	277	377	419	378	0.602	0.500	57	50
Camellia sasanqua	13.4	0.2	17.4	2.9	3	nd	nd	nd	nd	nd	nd	nd	nd
Podcarpus nagi	10.6		11.5		2	302	387	556	408	0.648	0.513	50	51
Rhododendron tashiroi	8.1		9.9		2	nd	nd	nd	nd	nd	nd	nd	nd
Myrica rubra	6.8	0.3	8.7	0.4	5	256	389	399	346	0.609	0.470	41	41
Quercus miyagii	6.8		9.4		2	nd	nd	nd	nd	nd	nd	nd	nd

[1] Each sample contained 10-50 leaf disks from 10-30 leaves.

Fig. S2. ^{13}C NMR spectra of bleached (BL) and nonbleached portions (NB) of leaf litter. (a) *Castanopsis sieboldii*, (b) *Distylium racemosum*, (c) *Persea thunbergii*, (d) *Schima wallichii*, (e) *Myrsine seguinii*, (f) *Ilex maximowicziana* var. *kanehirae*, (g) *Camellia japonica*, (h) *Elaeocarpus japonicus*, (i) *Meliosma squamulata*, (j) *Podocarpus nagi*, (k) *Rhododendron tashiroi*, (l) *Myrica rubra*.

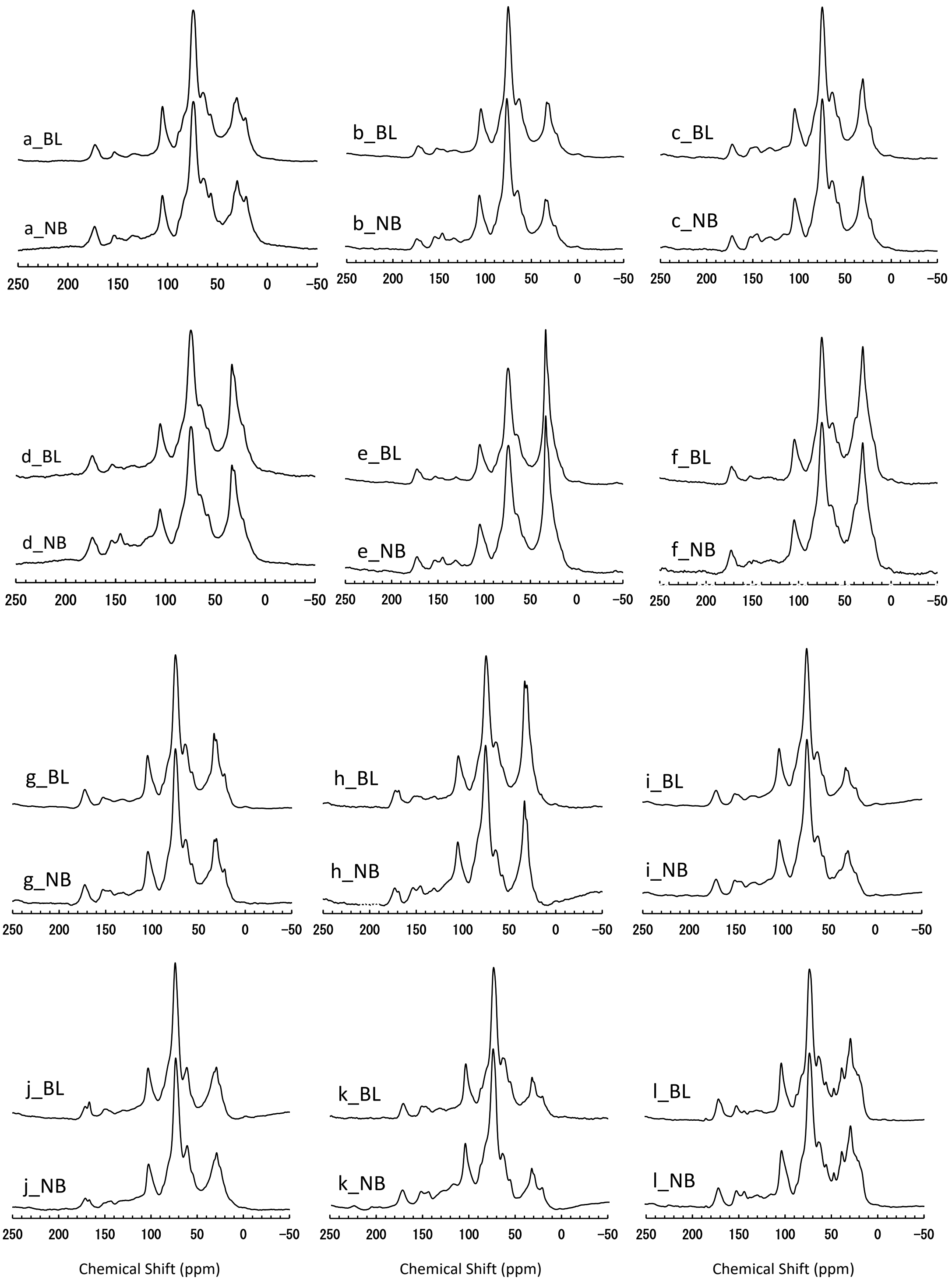

Table S3. Relative area (%) of ^{13}C NMR spectra in freshly fallen leaves (FF) and bleached (BL) and nonbleached portions (NB) of leaf litter for 12 plant species.

Plant species	Alkyl-C (0-45 ppm)			O-alkyl-C (45-110 ppm)			Aromatic-C (110-160 ppm)			Carbonyl-C (160-190 ppm)		
	FF	BL	NB	FF	BL	NB	FF	BL	NB	FF	BL	NB
Castanopsis sieboldii	22.7	25.3	26.0	64.8	64.7	60.9	9.3	6.7	9.4	3.1	3.3	3.7
Distylium racemosum	21.1	19.0	19.2	66.5	64.0	65.5	9.4	14.6	12.9	3.0	2.4	2.4
Persea thunbergii	24.3	21.5	22.4	62.5	63.3	62.0	10.5	13.3	13.1	2.6	1.9	2.5
Schima wallichii	27.0	30.4	26.0	57.3	58.0	54.1	12.6	8.0	15.4	3.1	3.6	4.5
Myrsine seguinii	37.1	29.4	35.2	52.7	56.7	51.1	6.9	10.9	10.7	3.3	3.0	3.0
Ilex maximowicziana var. *kanehirae*	39.1	26.8	32.7	52.2	62.3	53.5	5.9	7.7	10.2	2.9	3.1	3.6
Camellia japonica	23.8	18.5	22.3	62.8	62.7	60.6	9.7	14.8	13.0	3.7	4.0	4.0
Elaeocarpus japonicus	29.8	21.9	20.9	57.3	61.9	59.5	9.8	13.8	16.5	3.1	2.4	3.1
Meliosma squamulata	14.3	14.6	17.1	70.7	69.0	66.3	11.9	14.1	13.6	3.1	2.2	3.0
Podcarpus nagi	19.2	21.2	22.8	66.3	61.6	64.6	11.1	14.2	9.8	3.4	3.0	2.8
Rhododendron tashiroi	28.8	26.9	28.4	57.7	59.8	57.8	9.7	11.2	10.7	3.8	2.0	3.1
Myrica rubra	16.6	13.9	12.7	68.6	69.6	64.4	11.7	14.4	18.6	3.0	2.1	4.2

Table S4. Contents of components of dissolved nitrogen (μgN/g) of bleached (BL) and nonbleached portions (NB) of leaf litter for 13 plant species. TEN total extractable nitrogen, EON extractable organic nitrogen. %EON = EON / TEN × 100. N=3.

Plant species	TEN				EON				%EON				NH_4^+-N				NO_3^--N				NO_2^--N			
	BL		NB		BL		NB		BL		NB		BL		NB		BL		NB		BL		NB	
	Mean	se	Mean	se	Mean	se	Mean	se	Mean	se	Mean	se	Mean	se	Mean	se	Mean	se	Mean	se	Mean	se	Mean	se
Castanopsis sieboldii	701	109	382	49	648	109	337	46	92.2	1.0	88.0	0.8	51	2	44	4	0.5	0.1	0.5	0.1	1.1	0.1	0.9	0.1
Distylium racemosum	475	72	285	30	375	66	180	19	78.4	2.8	63.1	2.3	92	12	97	13	6.4	0.2	6.0	0.7	2.4	0.1	1.7	0.2
Schima wallichii	723	246	416	169	644	235	352	164	87.2	2.6	80.9	4.9	77	12	62	9	1.0	0.1	0.7	0.2	1.7	0.3	1.1	0.2
Myrsine seguinii	414	113	252	52	250	89	145	35	58.0	6.4	56.9	1.9	138	27	91	16	19.4	2.0	11.8	1.5	6.4	0.8	3.3	0.2
Ilex maximowicziana var. *kanehirae*	475	18	264	39	358	15	179	28	75.2	1.5	67.6	0.7	107	9	76	10	7.6	0.4	6.6	0.2	2.8	0.2	2.3	0.1
Camellia japonica	172	43	154	20	149	41	128	21	85.8	2.2	82.4	3.0	22	2	24	2	0.4	0.0	0.7	0.1	0.8	0.0	1.0	0.1
Elaeocarpus japonicus	392	91	346	32	277	68	219	20	70.4	0.8	63.6	3.7	98	23	116	18	13.3	0.4	8.7	1.1	3.3	0.4	2.0	0.0
Neolitsea sericea	465	122	206	24	343	109	129	20	70.2	7.2	62.1	2.3	99	19	55	4	17.3	2.7	16.1	0.8	6.0	1.0	5.8	0.3
Meliosma squamulata	630	9	418	38	382	43	257	38	60.5	6.1	60.9	3.6	230	37	141	3	13.3	1.3	15.2	1.7	4.3	0.6	4.8	0.5
Podcarpus nagi	663	11	350	19	555	19	286	17	83.6	1.8	81.7	1.2	97	10	44	4	8.0	0.8	15.2	0.7	2.9	0.1	4.4	0.2
Rhododendron tashiroi	590	81	313	12	474	71	213	8	80.2	1.6	67.8	0.5	96	11	82	6	14.3	2.1	14.4	1.0	4.5	1.0	4.2	0.2
Myrica rubra	1028	148	359	61	922	154	285	58	89.0	2.4	78.8	2.4	94	7	63	5	8.6	0.8	7.3	0.2	2.9	0.1	2.6	0.2
Quercus miyagii	617	49	328	14	484	47	238	7	78.3	1.7	72.5	0.9	113	7	75	8	15.0	0.2	11.7	1.2	4.8	0.0	4.0	0.4
